# CYLD Enhances Severe Listeriosis by Impairing IL-6/STAT3-Dependent Fibrin Production

**DOI:** 10.1371/journal.ppat.1003455

**Published:** 2013-06-27

**Authors:** Gopala Nishanth, Martina Deckert, Katharina Wex, Ramin Massoumi, Katrin Schweitzer, Michael Naumann, Dirk Schlüter

**Affiliations:** 1 Institute of Medical Microbiology, Otto-von-Guericke University Magdeburg, Magdeburg, Germany; 2 Department of Neuropathology, University Hospital Cologne, Cologne, Germany; 3 Department of Laboratory Medicine, Lund University, Malmö, Sweden; 4 Institute of Experimental Internal Medicine, Otto-von-Guericke University Magdeburg, Magdeburg, Germany; 5 Helmholtz Centre for Infection Research, Braunschweig, Germany; National Institute of Allergy and Infectious Diseases, National Institutes of Health, United States of America

## Abstract

The facultative intracellular bacterium *Listeria monocytogenes* (Lm) may cause severe infection in humans and livestock. Control of acute listeriosis is primarily dependent on innate immune responses, which are strongly regulated by NF-κB, and tissue protective factors including fibrin. However, molecular pathways connecting NF-κB and fibrin production are poorly described. Here, we investigated whether the deubiquitinating enzyme CYLD, which is an inhibitor of NF-κB-dependent immune responses, regulated these protective host responses in murine listeriosis. Upon high dose systemic infection, all C57BL/6 Cyld^−/−^ mice survived, whereas 100% of wildtype mice succumbed due to severe liver pathology with impaired pathogen control and hemorrhage within 6 days. Upon *in vitro* infection with Lm, CYLD reduced NF-κB-dependent production of reactive oxygen species, interleukin (IL)-6 secretion, and control of bacteria in macrophages. Furthermore, Western blot analyses showed that CYLD impaired STAT3-dependent fibrin production in cultivated hepatocytes. Immunoprecipitation experiments revealed that CYLD interacted with STAT3 in the cytoplasm and strongly reduced K63-ubiquitination of STAT3 in IL-6 stimulated hepatocytes. In addition, CYLD diminished IL-6-induced STAT3 activity by reducing nuclear accumulation of phosphorylated STAT3. *In vivo*, CYLD also reduced hepatic STAT3 K63-ubiquitination and activation, NF-κB activation, IL-6 and NOX2 mRNA production as well as fibrin production in murine listeriosis. *In vivo* neutralization of IL-6 by anti-IL-6 antibody, STAT3 by siRNA, and fibrin by warfarin treatment, respectively, demonstrated that IL-6-induced, STAT3-mediated fibrin production significantly contributed to protection in Cyld^−/−^ mice. In addition, *in vivo* Cyld siRNA treatment increased STAT3 phosphorylation, fibrin production, pathogen control and survival of Lm-infected WT mice illustrating that therapeutic inhibition of CYLD augments the protective NF-κB/IL-6/STAT3 pathway and fibrin production.

## Author Summary

*Listeria monocytogenes* causes high mortality in immunocompromised patients and fetuses. Murine studies have revealed that innate immune responses and fibrin, a major product of hepatocytes, are important to control *Listeria*. In the present study, we analysed whether the deubiquitinating enzyme CYLD impairs protective host responses in severe listeriosis and is a potential therapeutic target molecule. Using wildtype and Cyld^−/−^ mice, we show that CYLD significantly reduced pathogen control and production of interferon (IFN)-γ, interleukin (IL)-6, and NOX2 mRNA in liver and spleen resulting in death of wildtype but not of Cyld^−/−^ mice upon high-dose systemic infection. *In vitro*, CYLD impaired NF-κB-dependent pathogen control, reactive oxygen production, and IL-6 secretion in IFN-γ-stimulated, infected macrophages. We newly identified that CYLD directly removed K63-ubiquitin from STAT3, inhibited STAT3 activation and nuclear translocation resulting in reduced hepatocyte fibrin production. In *Listeria*-infected Cyld^−/−^ mice, hepatic STAT3 K63-ubiquitination and activation, NF-κB activation, IL-6 production, and fibrin deposition were also increased. Neutralization experiments confirmed that the improved survival and pathogen control of Cyld^−/−^ mice was dependent on IL-6-STAT3-mediated fibrin deposition. Finally, Cyld siRNA treatment of *Listeria*-infected wildtype mice significantly increased activated STAT3 and fibrin production, improved pathogen control and reduced mortality illustrating a therapeutic potential of CYLD inhibition.

## Introduction

*Listeria monocytogenes* (Lm) is a facultative intracellular, gram-positive rod, which may cause life threatening infections in the elderly (>65 years), immunocompromised patients and fetuses [1]. Clinically, listeriosis can present as septicaemia, disseminated inflammatory granuloma (granulomatosis infantiseptica), gastroenteritis, and focal infections including hepatitis as well as meningoencephalitis. Murine listeriosis is widely used as model disease to study the pathogenesis of human listeriosis and basic mechanisms of host - pathogen interactions. Ten minutes after i.v. infection, 60% of *Listeria* can be recovered from the liver and, after 6 hours, 95% of hepatic *Listeria* reside within hepatocytes [2]. Resistance to infection is dependent on an effective control of *Listeria* and requires the production of various cytokines and immune mediators including IFN-γ, TNF, IL-2, IL-6, IL-17, and the NOX2 (gp91^phox^, nicotine adenine dinucleotide phosphate oxidase)-dependent production of reactive oxygen species (ROS) [3]–[10], whereas IL-4 is associated with disease progression [11]. IFN-γ is essential for survival of acute systemic murine listeriosis and activates macrophages, which kill *Listeria* by a NOX2-dependent mechanism [9], [12]. In the liver, IL-6, which is mainly produced by local macrophages, i.e. Kupffer cells, induces STAT3 activation in hepatocytes and protects by inducing neutrophilia [13]. In addition to pro-inflammatory cytokines, immunosuppressive cytokines, in particular IL-10, are important to prevent lethal immunopathology, especially in cerebral listeriosis [14].

In addition to immune responses, fibrin is protective in listeriosis by restraining bacterial growth, suppressing hemorrhage, and pathology [15]. The molecular mechanisms regulating fibrin production in infectious diseases are largely unknown. Lim et al. [16] demonstrated that the deubiquitinating enzyme (DUB) CYLD inhibited p38 kinase-dependent expression of plasminogen activator inhibitor (PAI)-1 in murine lethal *Streptococcus pneumoniae* pneumonia. PAI-1 is required to prevent bacterial dissemination and alveolar hemorrhage. Since PAI-1 inhibits plasminogen production and fibrinolysis, the indirect inhibition of PAI-1 by CYLD in combination with reduced lung hemorrhage and increased PAI-1 production of Cyld^−/−^ mice indicate that CYLD caused augmented fibrinolysis. However, it remains unknown whether CYLD also regulates fibrin expression and deposition in addition to fibrinolysis.

CYLD is a tumor suppressor gene which is mutated in familial cylindromatosis, a disease characterized by benign tumors of the skin appendage [17]. In addition, expression of CYLD is down-regulated in several other types of human tumors including hepatocellular carcinoma, melanoma, colon cancer, and multiple myeloma [18]–[21]. CYLD has a high specificity in cleaving K63-linked polyubiquitin chains. Unlike K48-ubiquitin chains, which target proteins for proteasomal degradation, K63-ubiquitin chains exert non-degradative functions including modification of protein trafficking, protein-protein interactions and signal transduction [17]. Consequently, CYLD terminates the K63-dependent activity of several molecules including transforming growth factor β–activated kinase 1 (TAK1), TNF receptor-associated factor-2 (TRAF2), TRAF6, receptor-interacting protein-1, NF-κB essential modulator, c-Jun amino terminal kinase, retinoic acid-inducible gene-I, B cell leukemia-3 (Bcl-3) and p38 [22]. Consequently, CYLD inhibits the activation of the transcription factor NF-κB, which plays an important role for immune responses. To study the *in vivo* function of CYLD, four different Cyld^−/−^ mice have been developed and all of these mouse strains [16], [23], [24] except one [25] have a normal immune system. *In vivo*, the augmented NF-κB-dependent inflammatory reaction of Cyld^−/−^ mice resulted in a lethal *Escherichia coli* pneumonia and more severe *Haemophilus influenzae* middle ear infection [26], [27], whereas increased activation of p38 protected Cyld^−/−^ mice from lethal acute lung injury induced by *Streptococcus pneumoniae* infection [16].

In the present study, we used Cyld^−/−^ mice with a normal immune system and demonstrate that CYLD prevented survival from severe systemic listeriosis by (i) inhibiting protective NF-κB-dependent innate immune responses, (ii) impairing IL-6-induced STAT3 activation due to deubiquitination of K63-ubiquitinated STAT3 and (iii) reduction of STAT3-dependent fibrin production.

## Results

### CYLD prevented survival from severe listeriosis and aggravated liver pathology in listeriosis

**Figure 1:**
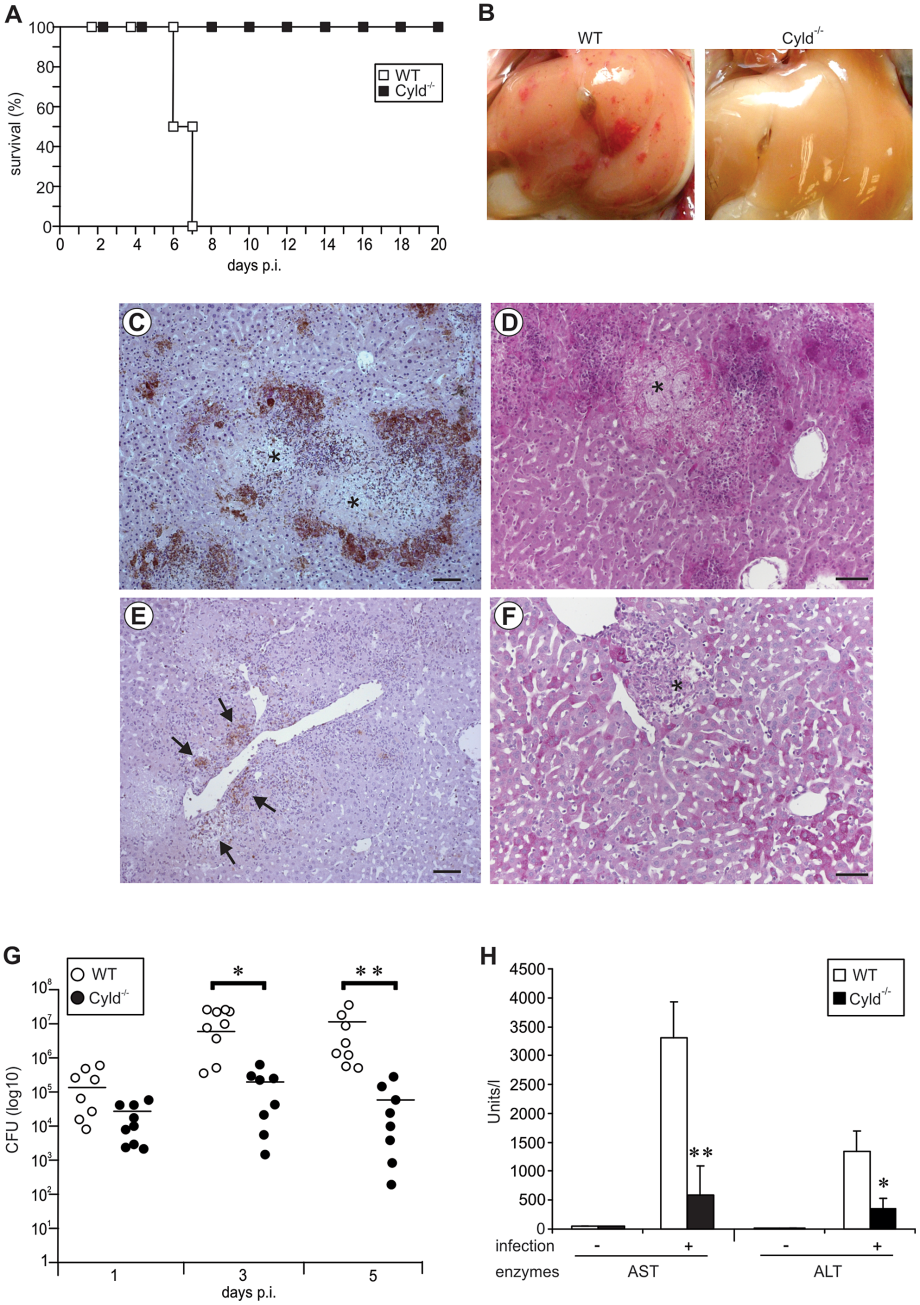
Cyld^−/−^ mice are protected from lethal listeriosis and severe liver pathology. (A) C57BL/6 Cyld^−/−^ (n = 10) and WT (n = 10) mice were infected i.v. with 5×10^5^ Lm and survival rates were monitored until day 20 p.i. (p<0.005 for WT vs. CYLD^−/−^ mice). One of two representative experiments is shown. (B) A macroscopic examination of livers from WT and Cyld^−/−^ mice showed severe haemorrhage in WT but not in Cyld^−/−^ mice at day 5 p.i. (C–F) Histopathology of WT (C, D) and Cyld^−/−^ mice (E, F) at day 5 p.i. (C, E) Immunohistochemistry with α-Lm antiserum in a WT (C) and Cyld^−/−^ mouse (E) (slight counterstaining with hemalum, bar 5 µm). In (C), * marks necrosis surrounded by clusters of Lm. (D, F) PAS staining of a WT (D) and Cyld^−/−^ (F) mouse (bar 10 µm). In (D), * indicates a large area of necrosis. In (F), * indicates a well-defined inflammatory focus. In (B–F), three mice per group were analysed and representative data are shown. The experiment was performed twice. (G) CFUs were determined in the liver of Lm-infected WT and Cyld^−/−^ mice at the indicated time points p.i. (* p<0.05, ** p<0.01). Data show the combined results of two independent experiments with a total of 8–9 mice per experimental group and time point. The mean of each experimental group is shown by a bar and each symbol represents one mouse. (H) The liver enzymes aspartate transaminase (AST) and alanine transaminase (ALT) were determined in serum at day 5 p.i. (* p<0.05 and ** p<0.01 for WT vs. Cyld^−/−^ mice). Data show the mean ± SD of 5 mice per experimental group from one of two representative experiments.

To investigate the functional role of CYLD in severe listeriosis, WT and Cyld^−/−^ mice were i.v. infected with 5×10^5^ CFU of Lm. Whereas allWT mice succumbed up to day 7 post infection (p.i.), 100% of Cyld^−/−^ mice survived (Fig. 1A).

At day 5 p.i., critically ill WT mice showed macroscopically severe liver hemorrhage, which was absent in Cyld^−/−^ mice (Fig. 1B). In WT mice, hepatic inflammation was widespread with ill-defined borders of the inflammatory infiltrates and large areas of necrosis were present (Fig. 1C). In addition, numerous Lm in huge, partially confluent inflammatory infiltrates were scattered throughout the liver of WT mice, being particularly prominent at the border of necroses (Fig. 1C). Lm and inflammatory infiltrates contributed to widespread necroses and total loss of glycogen from hepatocytes (Fig. 1D). In contrast to WT mice, necrosis was consistently absent from the liver of Cyld^−/−^ mice (Fig. 1E, F). In addition, Cyld^−/−^ mice harboured remarkably lower numbers of bacteria confined to well delineated granulomas of moderate size (Fig. 1E), which were associated with a focal loss of glycogen confined to the inflammatory lesions (Fig. 1F).

The improved pathogen control of Cyld^−/−^ mice was confirmed by determination of CFU, which revealed significantly lower numbers of Lm in the liver of Cyld^−/−^ mice at days 3 and 5 p.i. (Fig. 1G). In addition, the more severe liver pathology of WT mice also resulted in disturbance of liver function as revealed by increased serum alanine transaminase (ALT) and aspartate transaminase (AST) levels in Lm-infected WT mice (Fig. 1H).

### CYLD impaired IL-6, IFN-γ and NOX2 mRNA production and recruitment of myeloid cells to the liver

**Figure 2:**
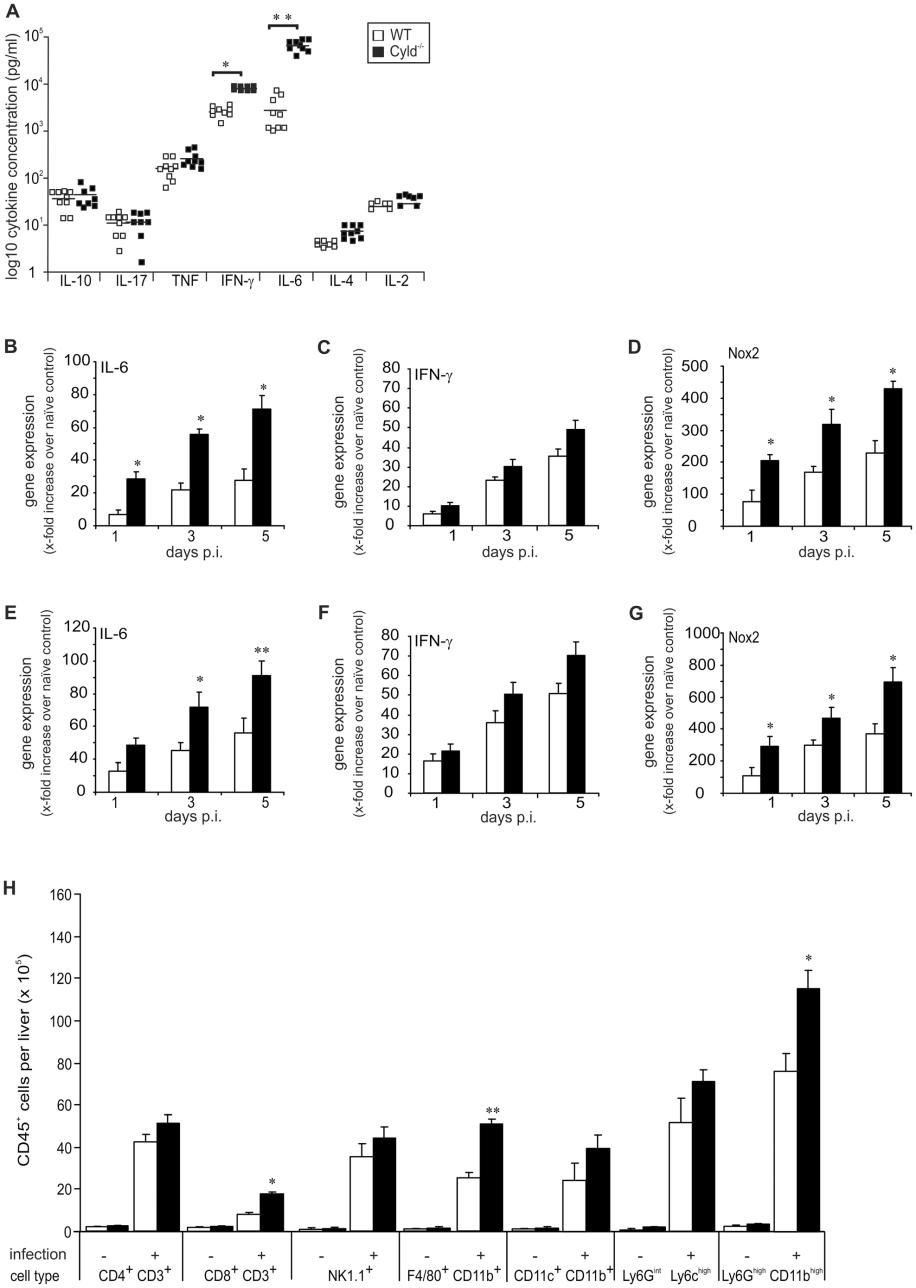
CYLD impairs IL-6 and IFN-γ production and leukocyte recruitment in listeriosis. (A) The serum concentrations of IL-10, IL-17, TNF, IFN-γ, IL-6, IL-4, and IL-2 were determined in Lm-infected WT and Cyld^−/−^ mice by a cytometric bead assay at day 5 p.i. (* p<0.05, ** p<0.01). Symbols represent individual mice from two representative experiments. (B–G) Quantitative RT-PCR analysis of hepatic (B–D) and splenic (E–G) IL-6, IFN-γ, and NOX2 mRNA expression. Data show the increase of the respective mRNA expression of Lm-infected over uninfected mice of the same mouse strain. Data represent the mean ± SD of 5 mice. Data from of two representative experiments are shown. (H) The number of different leukocyte populations was determined in cells isolated from livers of uninfected and Lm-infected WT and Cyld^−/−^ mice. Data show the mean ± SD of CD45^+^ cell populations from 5 mice per experimental group. Data from one of two representative experiments are shown (* p<0.05 and ** p<0.001 for WT vs. Cyld^−/−^ mice).

To explore the influence of CYLD on cytokine production in listeriosis, serum cytokine levels were determined at day 5 p.i. Levels of IL-6 and IFN-γ were significantly increased in Cyld^−/−^ mice, whereas serum levels of IL-2, IL-4, IL-10, IL-17, and TNF did not differ between the two mouse strains (Fig. 2A). To further analyse differences in IL-6 and IFN-γ, mRNA expression of these cytokines was analysed by quantitative RT-PCR in the liver and spleen. Both IL-6 and IFN-γ mRNA were up-regulated in livers (Fig. 2B, C) and spleens (Fig. 2E, F) of infected WT and Cyld^−/−^ mice but only IL-6 mRNA was significantly higher in Cyld^−/−^ as compared to WT mice. In addition, NOX2 mRNA was significantly higher expressed in liver and spleen of Lm-infected Cyld^−/−^ mice (Fig. 2D, G).

Since the protective action of IL-6 in listeriosis includes the recruitment of myeloid cells [7], the cellular composition of hepatic inflammatory infiltrates was determined in Cyld^−/−^ and WT mice at day 5 p.i. Flow cytometry revealed a significant increase of Ly6G^high^ CD11b^high^ granulocytes, F4/80^+^ CD11b^+^ macrophages and CD8^+^ CD3^+^ T cells in livers of Lm-infected Cyld^−/−^ mice (Fig. 2H, Fig. S1).

### CYLD reduced IL-6, ROS production and killing of Lm in *Listeria*-infected macrophages by impairing NF-κB activation

**Figure 3:**
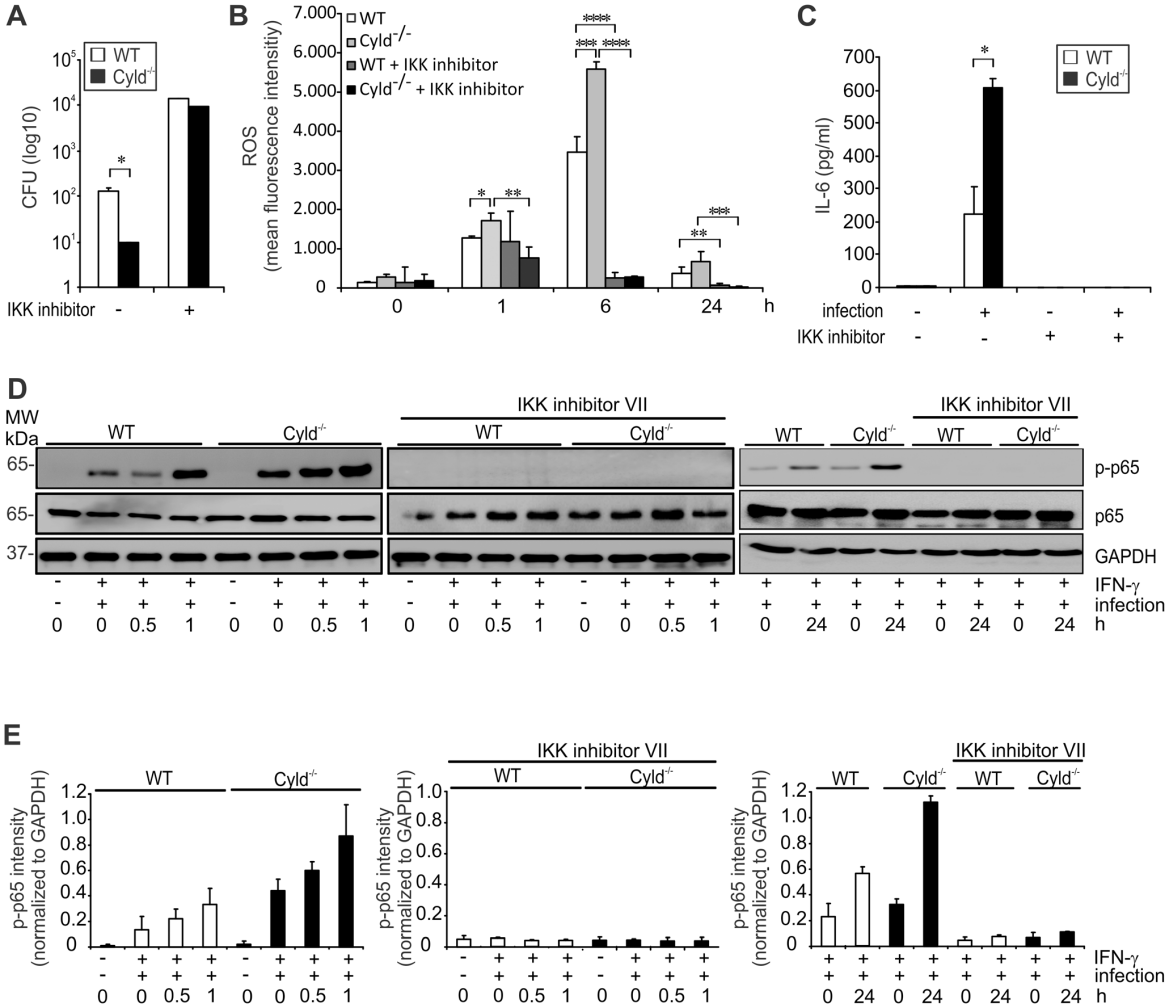
CYLD diminishes NF-κB-dependent IL-6 production, ROS production and pathogen control of Lm-infected macrophages. (A–C) BMDM were isolate from WT and Cyld^−/−^ mice and stimulated with IFN-γ (100 U/ml). Indicated groups were infected with Lm (MOI of 5∶1) and treated with IKK inhibitor (10 µM for 4 h followed by 1 µM for 20 h), respectively. (A) After 24 h, the amount of intracellular Lm was determined in 1×10^6^ BMDM. (B) ROS production was analysed by flow cytometry in Lm-infected macrophages 24 h after infection. (C) The supernatant was harvested from uninfected and infected macrophages after 24 h and analysed for IL-6 by CBA. In (A–C), data show the mean ± SD of triplicate wells; * p<0.05, ** p<0.01, *** p<0.005, **** p<0.001. (D) Proteins were isolated from uninfected and Lm-infected BMDM at the indicated time points. Cells were stimulated with IFN-γ and IKK inhibitor VII as indicated. WBs were incubated with α-p-p65, α-p65, and α-GAPDH as loading control. Representative WBs from a total of three independent experiments are shown. (E) Quantification of p-p65 intensity (± SD) was performed from WB data of uninfected and Lm-infected BMDM, which were stimulated as described in (D). The results present pooled data from 3 independent experiments.

To further study the impact of CYLD on IL-6 production and anti-bacterial activity, IFN-γ-stimulated bone marrow-derived macrophages (BMDMs) were infected with Lm. After 24 h of infection, CFUs were significantly reduced in Cyld^−/−^ macrophages (Fig. 3A), which correlated with a significantly increased production of ROS (Fig. 3B). In addition, IL-6 production was significantly increased in Cyld^−/−^ macrophages (Fig. 3C). Lm-infected IFN-γ-stimulated Cyld^−/−^ macrophages showed an enhanced activation of NF-κB mediated by an increased phosphorylation of p65 (Fig. 3D, E), which persisted until 24 h p.i. (Fig. 3D, E). Importantly, the improved pathogen control as well as production of ROS and IL-6 of Cyld^−/−^ macrophages was dependent on NF-κB, since inhibition of NF-κB activity by an IKK inhibitor abolished these protective macrophage responses (Fig. 3A–C).

### CYLD impaired IL-6-mediated STAT3 activation and fibrin production in hepatocytes by deubiquitination of cytoplasmic STAT3

**Figure 4:**
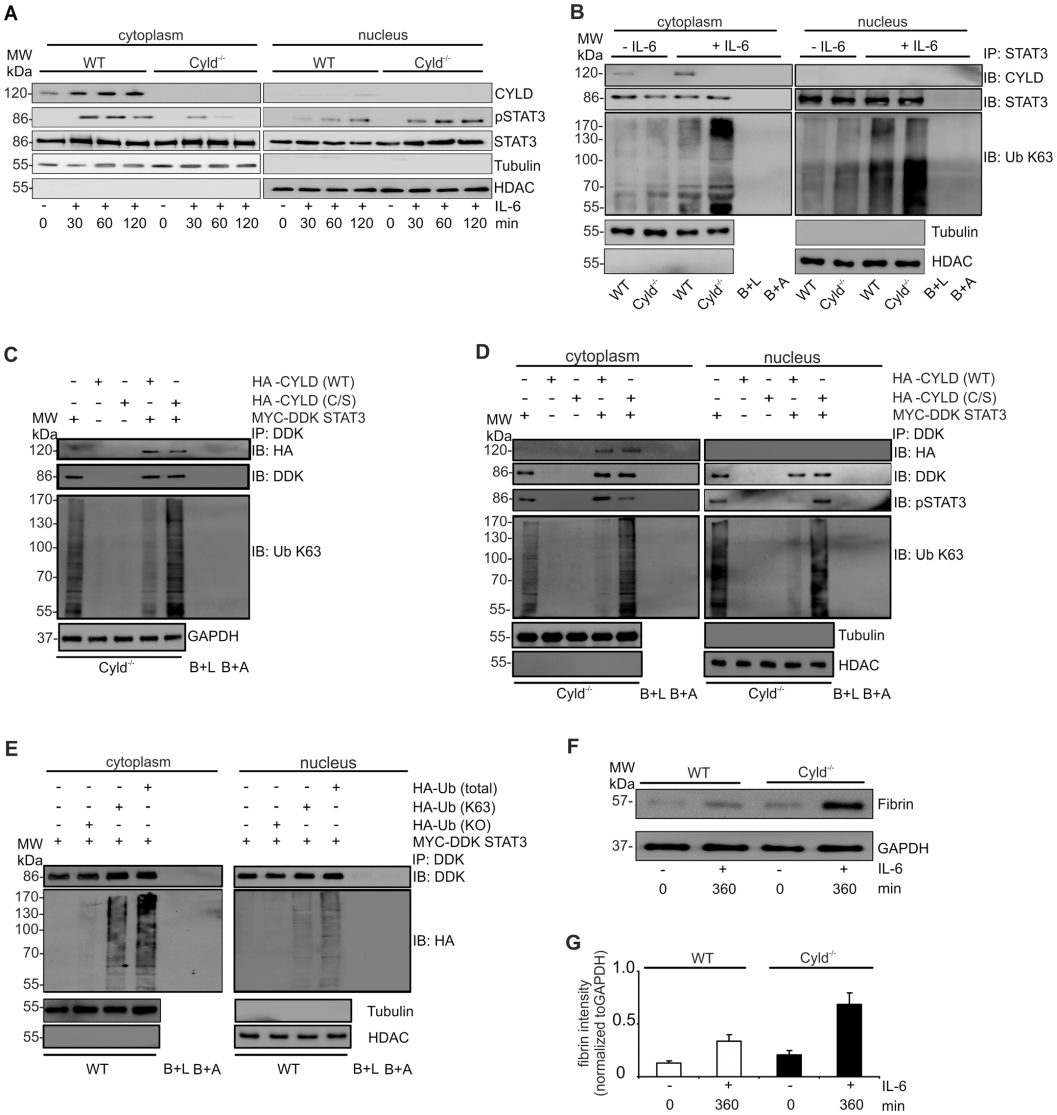
CYLD binds to STAT3 and inhibits nuclear accumulation of activated STAT3 and fibrin production in hepatocytes. (A) Proteins were isolated from the cytoplasm and nucleus, respectively, of WT and Cyld^−/−^ hepatocytes. WB were stained with α-tubulin and α-histone deacetylase (HDAC) as marker proteins for the cytoplasm and nucleus, respectively. (B) Cytoplasmic and nuclear protein lysates of unstimulated and IL-6-stimulated (200 ng/ml) WT and Cyld^−/−^ hepatocytes were immunoprecipitated with STAT3. Immunoprecipitates were stained for CYLD, STAT3, and K63-linked ubiquitin. The purity and amount of cytoplasmic and nuclear proteins was controlled by staining lysates for tubulin and HDAC, respectively, before immunoprecipitation. Beads plus lysate without antibody before immunoprecipitation (B+L) and beads plus STAT3 antibody without lysates (B+A) were used as controls. (C, D) Cyld^−/−^ hepatocytes were transfected with MYC-DDK STAT3, HA-CYLD (WT), mutant HA-CYLD (C/S) lacking catalytic activity as indicated. After IL-6 stimulation (1 h), total (C), cytoplasmic (D) and nuclear (D) lysates were immunoprecipitated with α-DDK. Immunoprecipitates were stained for the indicated proteins. (E) WT hepatocytes were transfected with MYC-DDK STAT3, ubiquitin with all lysine residues (HA-Ub total), ubiquitin with K63 only (HA-Ub K63), and ubiquitin with no lysine residues (HA-Ub KO) as indicated. After IL-6 stimulation (1 h), cytoplasmic and nuclear proteins were isolated and immunoprecipitated with α-DDK. Immunoprecipitates were stained for the indicated proteins. (F) WB analysis of fibrin production in unstimulated (0 min) and IL-6 stimulated (360 min) cultivated WT and Cyld^−/−^ hepatocytes. (G) Quantification of fibrin (± SD) was performed from WB data of unstimulated and IL-6-stimulated WT and Cyld^−/−^, respectively, hepatocytes. Representative results from one of three experiments are shown.

Since Lm-infected WT mice suffered from hemorrhage, we studied the impact of CYLD on IL-6-induced STAT3 activation and fibrin production in hepatocytes. IL-6 treatment resulted in an increase of CYLD protein in the cytoplasm of WT mice (Fig. 4A). Further, stimulation of WT and Cyld^−/−^ hepatocytes with IL-6 resulted in phosphorylation of cytoplasmic STAT3 (Fig. 4A). Within 60 min after stimulation, pSTAT3 declined in the cytosol of Cyld^−/−^ but not in WT hepatocytes (Fig. 4A). At 120 min, pSTAT3 was undetectable in the cytoplasm of Cyld^−/−^ hepatocytes, whereas it was still present in the cytoplasm of WT hepatocytes (Fig. 4A). In addition, IL-6-stimulation induced translocation of pSTAT3 to the nucleus of hepatocytes from both mouse strains. However, nuclear pSTAT3 amounts were much higher in Cyld^−/−^ as compared to WT hepatocytes 60 and 120 min after stimulation (Fig. 4A). In contrast to pSTAT3, non-phosphorylated STAT3 was found constitutively in the cytoplasm and nucleus of WT and Cyld^−/−^ hepatocytes (Fig. 4A), which is in accordance with the observation that importin-α3 constitutively shuttles non-phosphorylated STAT3 between cytoplasm and nucleus [28].

To study whether CYLD may regulate nuclear accumulation of pSTAT3 by deubiquitination of STAT3, we immunoprecipitated STAT3 from WT and Cyld^−/−^ hepatocytes. Western blot (WB) analysis of immunoprecipitates detected STAT3 in WT and Cyld^−/−^ hepatocytes, whereas CYLD was only detectable in WT hepatocytes (Fig. 4B). IL-6 stimulation induced a strong increase of K63-ubiquitination in Cyld^−/−^ hepatocytes but only a slight increase in IL-6-stimulated WT hepatocytes. Importantly, the amount of K63-ubiquitinated proteins was strongly augmented in immunoprecipitates of IL-6-stimulated Cyld^−/−^ hepatocytes as compared to WT hepatocytes suggesting that CYLD reduced K63-ubiquitination of STAT3 (Fig. 4B). In immunoprecipitates of nuclear STAT3, CYLD was undetectable further indicating that CYLD interacted with STAT3 in the cytosol (Fig. 4B).

Transfection of Cyld^−/−^ hepatocytes with MYC-DDK STAT3, HA-WT CYLD (CYLD WT), and catalytically inactive CYLD (CYLD C/S) followed by immunoprecipitation of STAT3 further showed that WT CYLD reduced K63-ubiquitination of STAT3 (Fig. 4C, total lysates). Catalytically inactive CYLD still interacted with STAT3 but failed to reduce K63-ubiquitination of STAT3 (Fig. 4C, D). STAT3 and CYLD interacted in the cytoplasm but not in the nucleus of IL-6-stimulated hepatocytes (Fig. 4D). The amount of nuclear pSTAT3 was strongly increased in hepatocytes transfected with catalytically inactive CYLD (Fig. 4D). Further analysis of the interaction between ubiquitin and STAT3 showed that ubiquitin with only K63 (HA Ub (K63)) bound efficiently to STAT3 in IL-6-stimulated WT hepatocytes, whereas ubiquitin with no lysine residues (HA-Ub (KO)) failed to interact with STAT3 (Fig. 4E).

Since IL-6 induces STAT3-dependent fibrin synthesis by hepatocytes [29], we studied the CYLD-dependent regulation of IL-6-induced fibrin production in cultivated WT and Cyld^−/−^ hepatocytes. Interestingly, IL-6-induced fibrin synthesis was strongly enhanced in Cyld^−/−^ as compared to WT hepatocytes (Fig. 4F, G). Thus, IL-6 induced up-regulation of CYLD expression and promoted deubiquitination of K63-ubiquitinated STAT3, which limited the amount of nuclear pSTAT3, thereby, reducing fibrin production.

### CYLD reduced activation of p65, JAK2, STAT3, and p38 MAPK as well as fibrin production in livers of *Listeria*-infected WT mice

**Figure 5:**
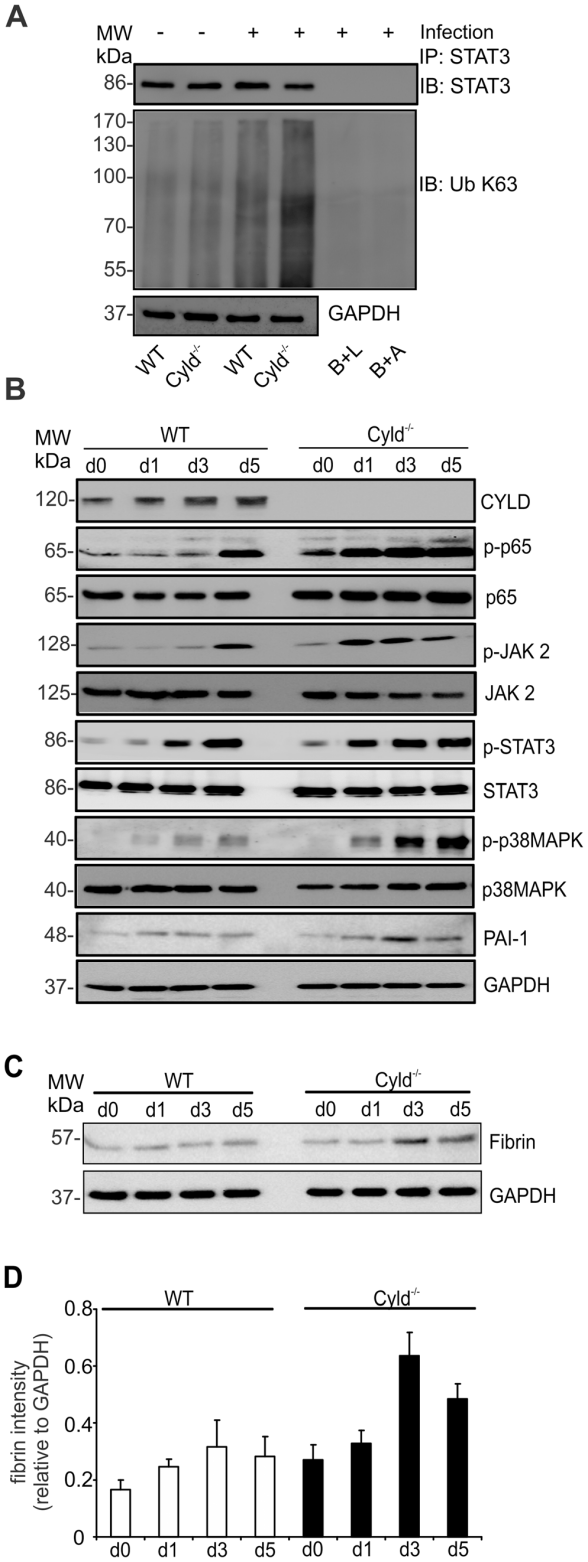
Reduced activation of p65, JAK2, STAT3, p38 MAPK and fibrin production in livers of *Listeria*-infected WT mice. (A) Proteins were isolated from livers of uninfected (d0) and Lm-infected WT and Cyld^−/−^ mice (6 h p.i.). Protein lysates were immunoprecipitated with STAT3 and WB was performed for STAT3 and K63-linked ubiquitin. (B, C) Proteins were isolated from livers of uninfected (d0) and Lm-infected WT and Cyld^−/−^ mice at days 1, 3, and 5 p.i. WB were incubated with α-CLYD, α-p-p65, α-p65, α-pSTAT3, α-STAT3, α-p-p38MAPK, α-p38MAPK, and α-PAI-1 (B) and fibrin (C). GAPDH was used as loading control. Three to four mice were analysed per group and representative data from one of two independent experiments are shown. (D) Quantification of fibrin (± SD) was performed from WB data of uninfected and Lm-infected WT and Cyld^−/−^, respectively. The results present 3 mice per group and time point.

In listeriosis, IL-6 production and STAT3 activation are initiated within the first hours p.i. (Gregory 1998). Therefore, we studied the impact of CYLD on STAT3 K63-ubiquitination in livers of Lm-infected mice 6 hours p.i. (Fig. 5A). Infection with Lm increased K63-ubiquitination of STAT3, which was augmented in Cyld^−/−^ as compared to WT mice. Thus, CYLD regulated STAT3 ubiquitination also *in vivo*. In good agreement with an inhibitory role of CYLD on NF-κB activity in macrophages (Fig. 3D) and on STAT3 activation in hepatocytes (Fig. 4A, D), increased hepatic CYLD production of Lm-infected WT mice correlated with a reduced and delayed phosphorylation of p65, JAK2 and STAT3 as compared to Cyld^−/−^ mice (Fig. 5B). In addition, phosphorylation of p38 MAPK was reduced in livers of WT mice. Expression of PAI-1, which is induced by MAP kinases, was only slightly reduced in WT mice. Importantly, fibrin deposition was increased in Cyld^−/−^ as compared to WT mice at day 3 and 5 p.i. (Fig. 5C, D).

### The protection of Cyld^−/−^ mice against lethal listeriosis is dependent on IL-6, STAT3 and fibrin

**Figure 6:**
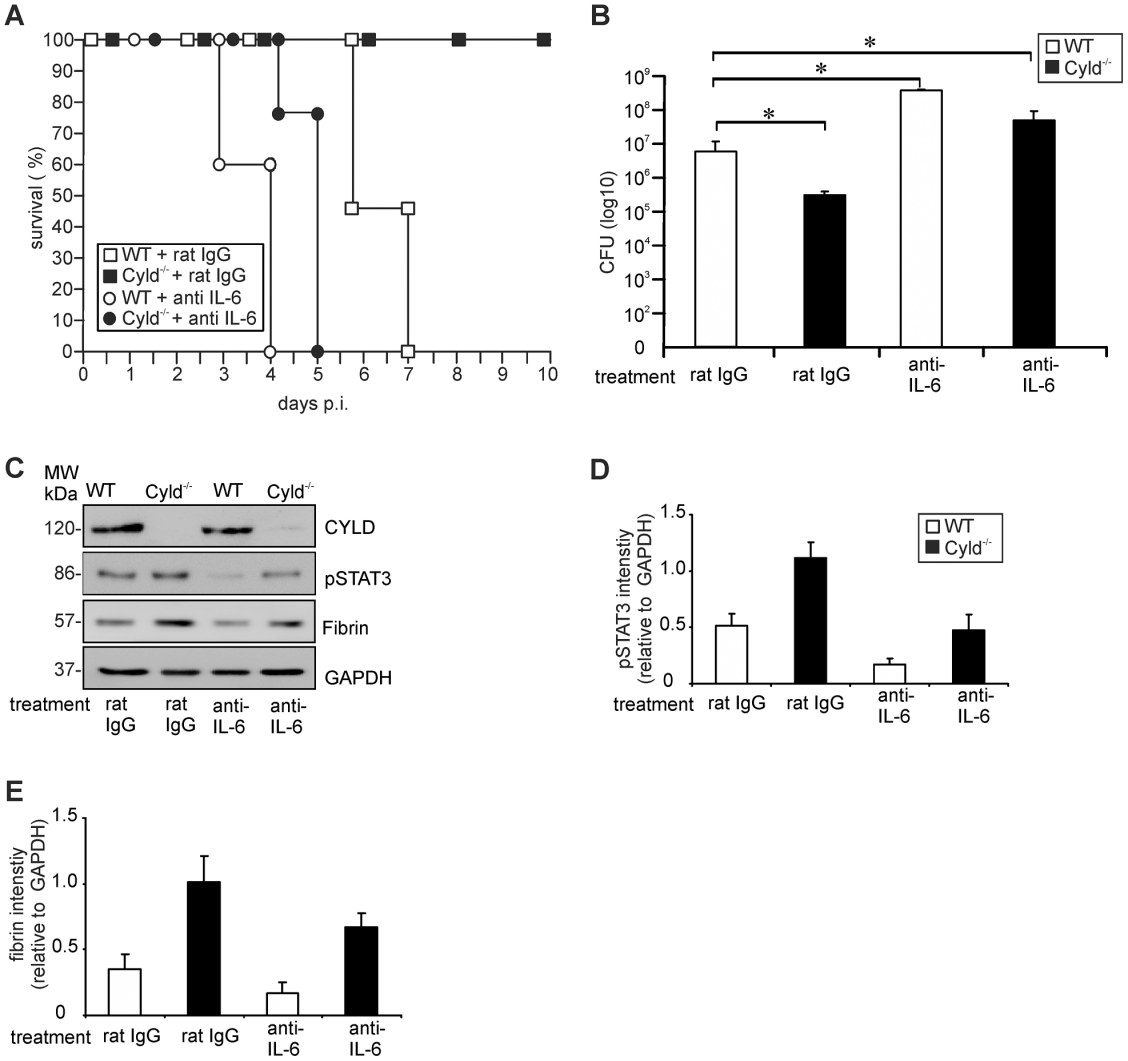
IL-6 neutralization abolishes increased STAT3 activation, fibrin production, survival and pathogen control in Lm-infected Cyld^−/−^ mice. (A) The survival rates of rat IgG and α-IL-6-treated Lm-infected WT and Cyld^−/−^ mice (n = 7 per experimental group) are shown. The survival rate of IgG-treated Cyld^−/−^ mice (p<0.05) but not of the other groups was significantly increased as compared to rat IgG-treated WT mice. (B) CFUs were determined in the liver of Lm-infected rat IgG and IL-6-treated WT and Cyld^−/−^ mice at day 5 p.i. (n = 5 per experimental group; * p<0.05). Data show the mean ± SD from one of two representative experiments. (C) Proteins were isolated from livers of infected rat IgG and IL-6-treated WT and Cyld^−/−^ mice (n = 3 per experimental group) at day 5 p.i. WB analysis for CYLD, pSTAT3, fibrin and GAPDH was performed and representative data are shown. (D, E). Quantification of hepatic pSTAT3 (D) and fibrin (E) (± SD) was performed from WB data of rat IgG and α-IL-6-treated WT and Cyld^−/−^ mice, respectively. The results present 3 mice of each experimental group.

To study whether the IL-6 induced STAT3 activation protected Cyld^−/−^ mice from lethal listeriosis by increased fibrin production, *in vivo* IL-6, STAT3 and fibrin neutralization experiments were performed. Neutralization of endogenous IL-6 by i.p. administration of the monoclonal MP5-20F3 antibody 1 h before i.v. Lm infection completely abolished the protective effect of Cyld-deficiency and all α-IL-6-treated Cyld^−/−^ mice succumbed up to day 5 p.i. (Fig. 6A). In addition, 100% of α-IL-6- and rat IgG-treated WT mice, respectively, succumbed, whereas all control antibody-treated Cyld^−/−^ mice survived (Fig. 6A). The improved pathogen control of Cyld^−/−^ mice was abolished by IL-6 neutralization and α-IL-6-treated Cyld^−/−^ mice had even significantly higher CFUs than rat IgG-treated WT mice at day 3 p.i. (Fig. 6B). Furthermore in Cyld^−/−^ mice, IL-6 neutralization resulted in reduction of hepatic pSTAT3 (Fig. 6C, D) and fibrin (Fig. 6C, E) as compared to rat IgG-treated Cyld^−/−^ mice. In WT mice, IL-6 neutralization only slightly reduced pSTAT3 without affecting fibrin (Fig. 6C, E) indicating that IL-6 induced pSTAT3 is strongly regulated by CYLD, which limits STAT3 activity and STAT3-dependent fibrin production.

**Figure 7:**
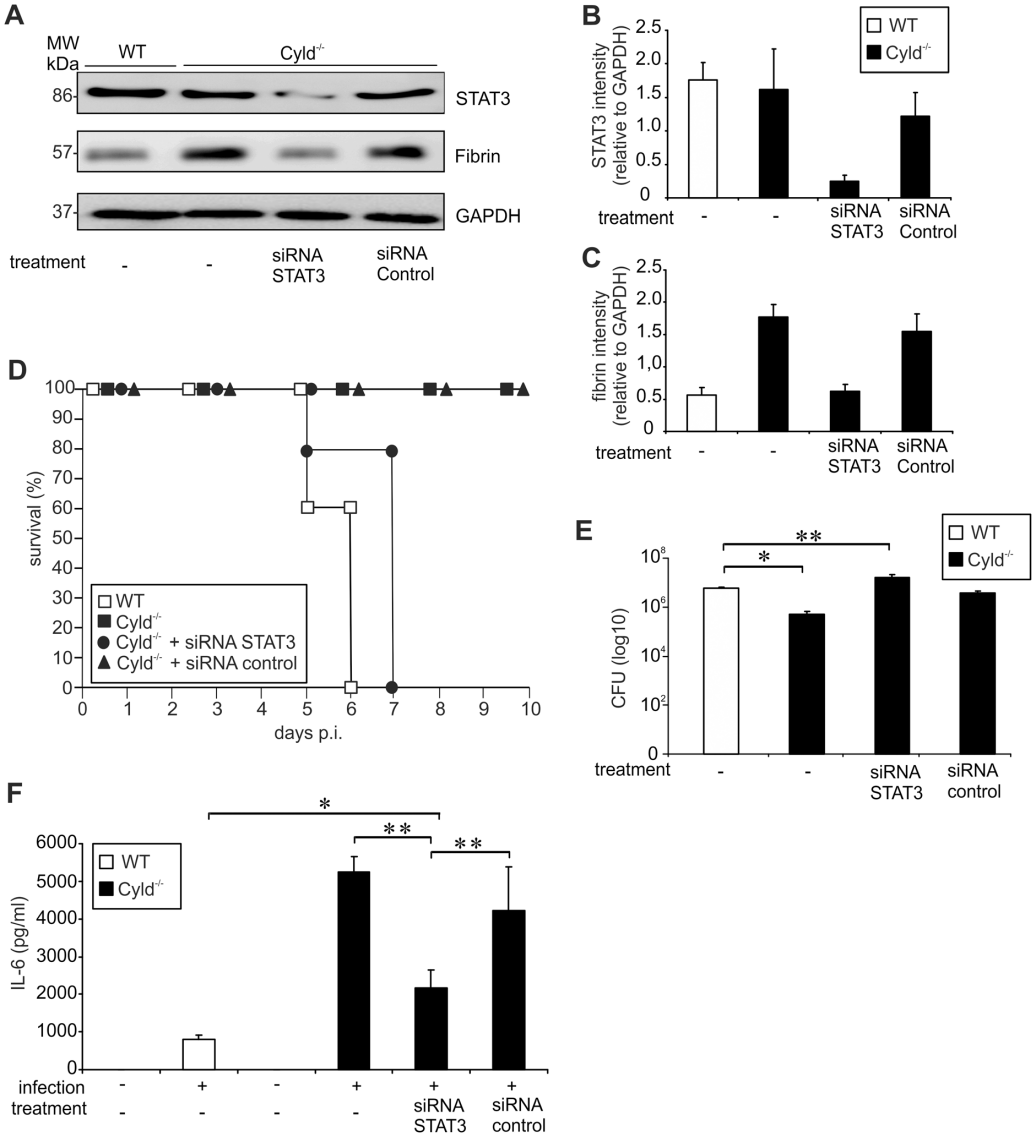
Inhibition of STAT3 reduces fibrin production, survival and pathogen control in Lm-infected Cyld^−/−^ mice. (A) Proteins were isolated from infected livers of untreated WT and Cyld^−/−^ mice as well as STAT3 siRNA and control siRNA-treated Cyld^−/−^ mice at day 5 p.i. (n = 3 experimental group). WB analysis for CYLD, pSTAT3, fibrin and GAPDH was performed and representative data are shown. (B, C) Quantification of total STAT3 (B) and fibrin (C) (± SD) was performed from WB data of livers from Lm-infected WT and Cyld^−/−^ mice, which were treated as indicated. The results present n = 3 mice per experimental group. (D) The survival rates of infected WT and Cyld^−/−^ as well as STAT3 siRNA and control siRNA-treated Cyld^−/−^ mice are shown. The survival of Cyld^−/−^ and control siRNA-treated Cyld^−/−^ mice but not of STAT3 siRNA-treated Cyld^−/−^ mice was significantly increased as compared to WT animals (p<0.05 for both groups, n = 5 per experimental group). Survival was monitored until day 10 p.i. One of two representative experiments is shown. (E) CFUs were determined in the liver of Lm-infected untreated WT and Cyld^−/−^ mice as well as STAT3 siRNA and control siRNA-treated Cyld^−/−^ mice at day 5 p.i. (* p<0.05, ** p<0.01; n = 5 per experimental group). Data show the mean ± SD and one of two representative experiments. (F) The serum concentration of IL-6 was determined by a cytometric bead assay at day 5 p.i. Data show the mean + SD of 5 mice per experimental group and from one of two representative experiments (* p<0.05, ** p<0.01).

To further study the impact of CYLD on IL-6-induced, STAT3-dependent fibrin production, STAT3 small interfering (si) RNA experiments were performed. I.v. treatment of Cyld^−/−^ mice with STAT3 siRNA 24 h before infection resulted in 80% reduction of total STAT3 in the liver (Fig. 7A, B), which caused reduced fibrin deposition in Cyld^−/−^ mice (Fig. 7A, C). In contrast, fibrin deposition was not reduced in control siRNA-treated and untreated infected Cyld^−/−^ mice (Fig. 7A, C). Importantly, STAT3 siRNA treatment induced 100% mortality of Lm-infected Cyld^−/−^ mice, thus, resembling WT mice (Fig. 7D). In contrast, control siRNA and untreated Cyld^−/−^ mice, respectively, survived the infection (Fig. 7D). Knockdown of STAT3 in Cyld^−/−^ mice also abolished the improved pathogen control of Cyld^−/−^ in comparison to WT mice (Fig. 7E). In contrast, control siRNA-treated and untreated Cyld^−/−^ mice had significantly lower CFUs as compared to WT mice (Fig. 7E) illustrating that siRNA-treatment did not unspecifically effect pathogen control. It has been reported that STAT3, in addition to NF-κB, contributes to IL-6 production [30]. Accordingly, knockdown of STAT3 significantly reduced serum IL-6 levels in Lm-infected Cyld^−/−^ mice as compared to mock-treated infected Cyld^−/−^ mice as well as untreated infected Cyld^−/−^ mice (Fig. 7F). In STAT3 siRNA-treated Cyld^−/−^ mice IL-6 levels were still increased as compared to untreated WT mice (Fig. 7F).

**Figure 8:**
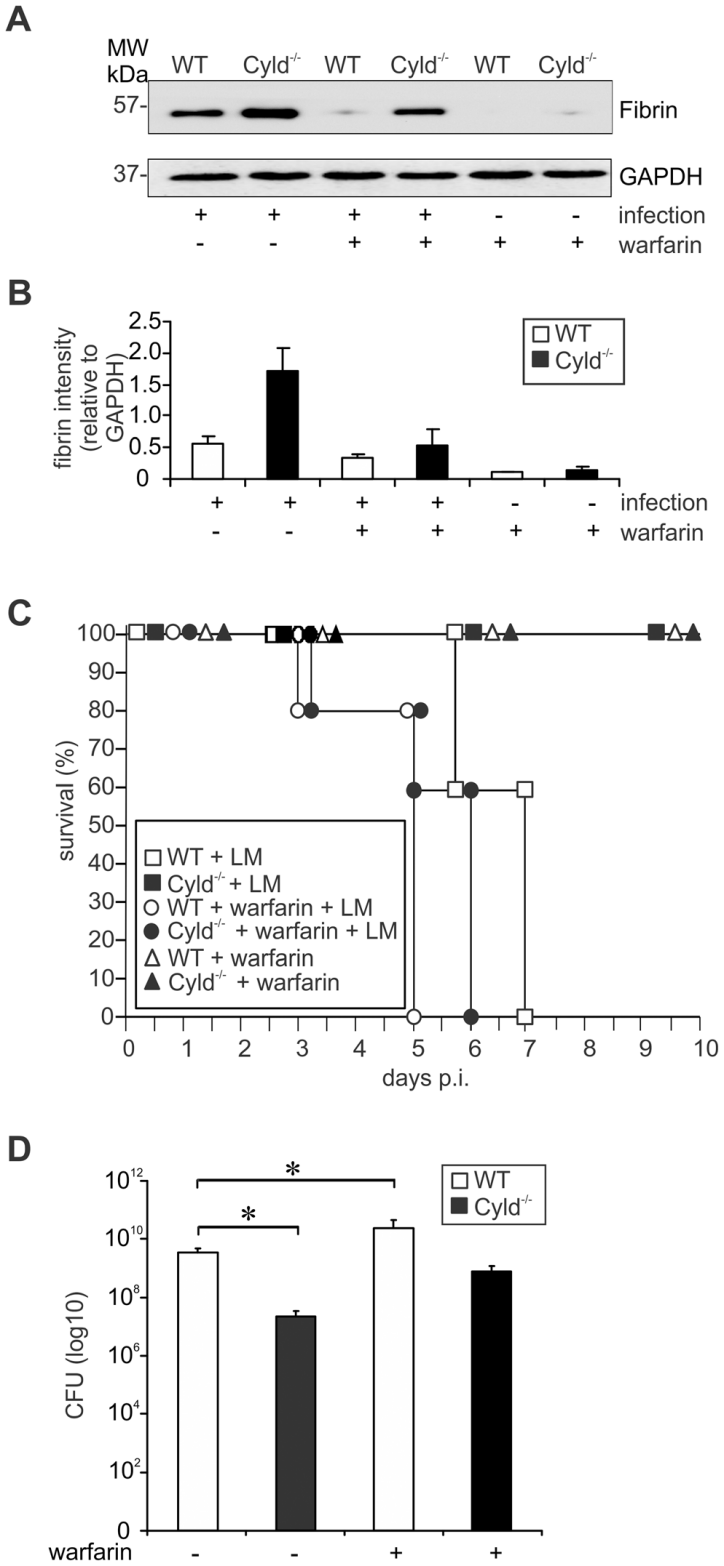
Inhibition of fibrin production abolished protection and increased the hepatic bacterial load of Cyld^−/−^ mice. (A) WB analysis of hepatic fibrin production in uninfected and infected WT and Cyld^−/−^ mice. GAPDH was used as loading control. (B) Quantification of fibrin (± SD) was performed from WB data of uninfected and Lm-infected WT and Cyld^−/−^, respectively, which were treated with warfarin as indicated. The results present 3 mice per experimental group. (C) The survival rate of uninfected and infected mice, which were treated with warfarin as indicated, was monitored until day 10 of infection (n = 10 per experimental group). Survival of infected Cyld^−/−^, uninfected Cyld^−/−^ mice treated with warfarin, and WT mice treated with warfarin, respectively, was significantly increased as compared to infected WT mice without warfarin treatment (p<0.001 for all groups). (D) CFUs were determined in the liver of Lm-infected WT and Cyld^−/−^ mice, which were treated with warfarin as indicated, at day 5 p.i. (* p<0.05, n = 5 per experimental group). Data show the mean ± SD. In (C) and (D) one of two representative experiments is shown.

To prove that the increased IL-6/STAT-3-dependent fibrin production protected Cyld^−/−^ mice from lethal listeriosis, fibrin deposition was inhibited by treatment with warfarin, a vitamin K antagonist. Warfarin treatment strongly reduced hepatic fibrin levels of both Lm-infected WT and Cyld^−/−^ mice (Fig. 8A, B). Suppression of fibrin production completely abolished protection from severe listeriosis in Lm-infected Cyld^−/−^ mice and warfarin-treated Cyld^−/−^ and WT mice died up to day 5 and 6 p.i., respectively (Fig. 8C). Untreated infected WT mice died later, i.e. until day 7 p.i., indicating that the low amounts of fibrin produced in WT mice still had a minor protective effect. Concomitantly, inhibition of fibrin production in WT mice resulted in increased hepatic CFUs as compared to non-treated WT animals (Fig. 8D). In Cyld^−/−^ mice, warfarin treatment resulted in an increase of hepatic CFUs, which were no longer significantly reduced as compared to untreated WT mice. Overall, α-IL6, STAT3 siRNA, and warfarin treatment further illustrated that CYLD impaired IL-6/STAT3-induced fibrin production resulting in impaired pathogen control and death from severe listeriosis.

### Inhibition of CYLD partially protected WT mice from lethal listeriosis

**Figure 9:**
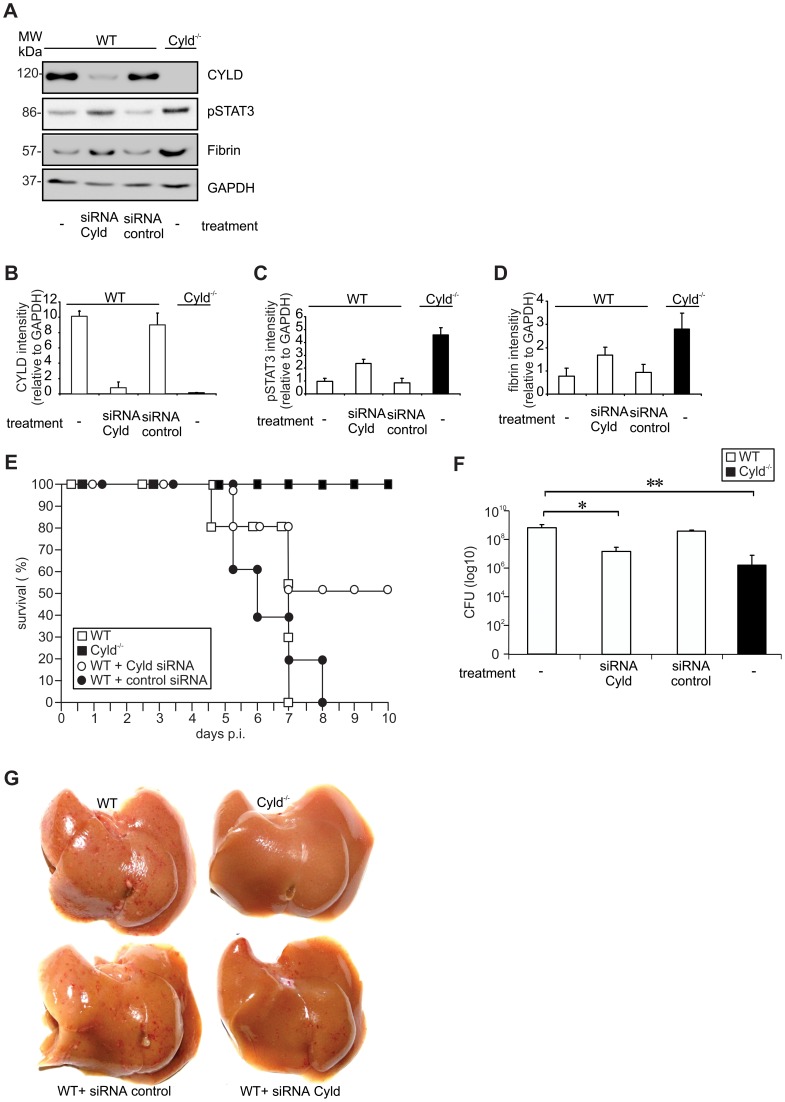
Therapeutic Cyld siRNA treatment protects WT mice from lethal listeriosis. (A) WT and Cyld^−/−^ mice were i.v. infected with 5×10^5^ Lm. Infected mice were treated as indicated 24 h p.i. At day 5 p.i., proteins were isolated from the liver (n = 3 per experimental group) and CYLD, pSTAT3 fibrin, and GAPDH production was analysed by WB. (B, C, D) Quantification of CYLD (B), pSTAT3 (C) and fibrin (D) (± SD) was performed from WB data of the indicated groups. The results present 3 mice per experimental group. (E) The survival rates of Lm-infected untreated Cyld^−/−^ and Cyld siRNA-treated WT mice were significantly were increased as compared to untreated WT mice (p<0.01 for Cyld^−/−^ vs. WT mice, p<0.05 for Cyld siRNA treated WT vs. WT mice). Ten mice per group were analysed until day 10 p.i. (F) CFUs were determined in the liver at day 5 p.i. Five mice were analysed per group and data show the mean ± SD (* p<0.05, ** p<0.01). In (A–G) data from one of two representative experiments are shown. (G) Macroscopic analysis showed haemorrhage of untreated and control siRNA-treated infected WT mice. Haemorrhage was reduced in WT mice treated with Cyld siRNA and was absent from untreated Cyld^−/−^ mice.

To explore whether knockdown of CYLD can protect WT mice from lethal listeriosis, WT mice were treated with Cyld siRNA 24 hours prior to Lm infection. WB analysis showed a 90% CYLD knockdown in livers of CYLD siRNA-treated WT mice, whereas treatment with control siRNA had no effect on CYLD protein levels of WT mice at day 5 p.i. (Fig. 9A, B). CYLD knockdown resulted in an increase of pSTAT3 (Fig. 9A, C) as well as fibrin (Fig. 9A, D) in Lm-infected WT mice. The increased pSTAT3 and fibrin levels of Cyld siRNA-treated, infected WT mice were associated with 50% survival, whereas all mock-treated and untreated WT mice succumbed (Fig. 9E). Thus, CYLD inhibition significantly reduced mortality of WT mice. Furthermore, siRNA-mediated CYLD inhibition significantly reduced CFUs in WT mice as compared to untreated and mock-treated WT animals, respectively (Fig. 9F). Macroscopic analysis of livers revealed strongly reduced hepatic hemorrhage in Cyld siRNA-treated WT mice as compared to untreated as well as mock-treated WT animals (Fig. 9G). In conclusion, these findings identify CYLD as a potential therapeutic target in severe listeriosis.

## Discussion

The present study shows that CYLD is a negative regulator of host survival in systemic listeriosis and that Cyld-deficiency protects mice from lethal listeriosis, hemorrhage and liver failure. Control of listeriosis is a complex process, which requires the synergistic activity of various innate immune mechanisms in combination with an appropriate activation of the coagulation system. Importantly, CYLD negatively interfered with several innate immune responses including IFN-γ production, NF-κB-dependent listericidal activity and IL-6 and ROS production of macrophages, and neutrophil recruitment to the infected liver, which all contribute to the control of Lm *in vivo* [3], [7], [9], [10], [13]. In addition, CYLD reduced K63-ubiquitination and phosphorylation of STAT3 resulting in an impaired IL-6/STAT3-dependent production of fibrin by hepatocytes as well as fibrin deposition in the liver, which are also important for protection in listeriosis. Importantly, CYLD diminished both NF-κB activation and K63-ubiquitination of STAT3 in livers of Lm-infected mice indicating that the combined inhibitory activity of CYLD on these signalling pathways resulted in a failure to control Lm and death of WT mice. However, CYLD did not suppress all protective host responses, and, for instance, production of IL-10, IL-17 and TNF, which all contribute to the defence against Lm [4], [5], [8], [14], were equally produced in WT and Cyld^−/−^ mice.

Only IFN-γ and IL-6 were significantly regulated by CYLD and increased in Cyld^−/−^ mice. IFN-γ is an important protective cytokine in listeriosis [3] and, thus, the improved course of disease in Cyld^−/−^ mice is partially explained by elevated IFN-γ production. In fact, *in vitro* infection of IFN-γ-stimulated BMDMs demonstrated that CYLD inhibited activity of NF-κB resulting in reduced IL-6 production, ROS synthesis, and bacterial killing, which were all dependent on NF-κB (Fig. 3). In good agreement, reduced p-p65, IL-6 mRNA, NOX2 mRNA, and increased CFUs were also present in livers of Lm-infected WT, and, thus the improved survival of Cyld^−/−^ mice was at least partially dependent on the augmented NF-κB activation. These data extend previous reports on the inhibitory function of CYLD on TRAF2, TRAF6, receptor-interacting protein-1, Bcl-3, and NF-κB essential modulator, which all contribute to NF-κB activation [23], [31]–[34]. An increased NF-κB activity was also observed in Cyld^−/−^ mice infected with the Gram-negative bacteria *Escherichia coli* and *Haemophilus influenzae* [26], [27]. Importantly, in these experimental models inhibition of NF-κB by CYLD was protective, which is in marked contrast to our study in listeriosis.

One of the protective NF-κB-dependent mechanisms in Cyld^−/−^ mice is the elevated IL-6 production. In listeriosis, the protective effect of endogenous IL-6, which is mainly produced by resident hepatic macrophages, i.e. Kupffer cells, includes a more effective control of Lm in the liver as well as increased neutrophilia [7]. In good agreement, we also observed that increased IL-6 production of Cyld^−/−^ mice upon high-dose Lm infection correlated with an augmented recruitment of neutrophils causing an improved control of Lm in the liver. Neutralization of IL-6 in otherwise protected Cyld^−/−^ mice resulted in failure to control Lm and death from listeriosis.

In extension, we newly identified that IL-6 does not only induce STAT3 phosphorylation in hepatocytes [13], but that CYLD strongly reduced IL-6-dependent accumulation of activated STAT3 in hepatic nuclei. In contrast, the amount of cytoplasmic pSTAT3 did not differ between WT and Cyld^−/−^ hepatocytes early after IL-6 stimulation indicating that the initial phosphorylation of STAT3 was not affected by CYLD. Of note, the amount of CYLD associated with STAT3 increased upon IL-6 stimulation (Fig. 4B). In hepatic listeriosis, Cyld-deficiency increased K63-ubiquitination of STAT3, and further *in vitro* experiments demonstrated that CYLD inhibited K63-ubiquitination of STAT3 in the cytoplasm of IL-6-stimulated hepatocytes, which is in agreement with the exclusive cytoplasmic localisation of CYLD. In addition, we identified that ubiquitin, in which only K63 was present, efficiently ubiquitinated STAT3. This observation extends recent data on TRAF6-dependent K63-ubiquitination of STAT3 [35]. In this study, TRAF6-mediated K63-ubiquitination of STAT3 inhibited IFN-α-induced STAT3-dependent expression of C-reactive protein and α-antichymotrypsin. In contrast, in our study increased K63-ubiquitination of STAT3 due to Cyld-deficiency induced increased STAT3-mediated fibrin production indicating that the underlying STAT3-activating stimulus is important. In fact, STAT3 can induce both proinflammatory immune reactions, e.g. upon stimulation with IL-6, but also mediate immunosuppressive functions, e.g. upon stimulation with IL-10 [36]. In our experiments, the inhibitory effect of CYLD on the nuclear accumulation of phosphorylated STAT3 resembles CYLD-Bcl3 interaction, since CYLD also deubiquitinates Bcl-3 in perinuclear regions and prevents its translocation to the nucleus, a process which significantly contributes to the development of keratinocyte hyperproliferation and development of benign tumors of the skin appendage called cylindromatosis [23].

In Cyld^−/−^ mice, both increased NF-κB-dependent IL-6 production and K63-ubiquitination of STAT3 contribute to the enhanced STAT3 activity, which further illustrates the central importance of IL-6/gp130-dependent STAT3 activity for protective host responses in infectious diseases [37], [38]. In extension to current knowledge, we uncover that the CYLD-dependent suppression of STAT3 activity in hepatocytes resulted in a decreased fibrin production, which identifies CYLD as an important regulator of IL-6-induced STAT3-dependent fibrin production. Hepatocytes are the most important cellular source of fibrinogen [39] and, herein, we show that the increased STAT3 activity in the liver of Lm-infected Cyld^−/−^ mice associated with an increased fibrin production (Fig. 5B). In addition to gp130/STAT3 signalling, IL-6 activates the gp130/SHP-2/MAPK pathway [40], [41]. In accordance, increased IL-6 levels of Lm-infected Cyld^−/−^ mice correlated with their elevated hepatic STAT3 and p38 MAPK activation. Activation of the IL-6/gp130/SHP2/MAPK pathway, which is also protective in listeriosis [42], induces p38-dependent PAI-1 production, which positively regulates fibrin deposition by inhibition of fibrinolysis. In Cyld^−/−^ mice, increased hepatic p-38 MAPK activity at days 3 and 5 p.i. correlated only with a slight increase of PAI-1 expression indicating that this pathway may contribute to a minor extent to the increased fibrin production in Lm-infected Cyld^−/−^ mice. An important negative impact of CYLD on p38-dependent protective PAI-1 induction has recently been shown in severe *Streptococcus pneumoniae*-induced acute lung injury [16]. In this model, combined activation of NF-κB and STAT3 plays an important role in the acute phase response, including fibrinogen production of hepatocytes and survival [43]. Thus, CYLD prevents survival of *S. pneumoniae* lung injury by combined inhibition of protective host responses in lung and liver.

Neutralization of fibrin formation by warfarin treatment completely abolished the protective effect of Cyld-deficiency on survival and impaired hepatic pathogen control. These findings extend previous observations of Mullarky et al. [15], who observed a protective function of fibrin on hepatic pathogen control and survival in murine listeriosis. A protective function of fibrin has also been observed in murine toxoplasmosis [44], in which fibrin inhibited immunopathology, as well as in *Yersinia enterocolitica* infection [45]. However, excessive fibrin production may also have deleterious consequences in infectious diseases. In particular, disseminated intravascular coagulation is a life-threatening complication in sepsis [46]. In this context it is of note that Lm is a facultative intracellular bacterium which mostly causes infections of organs. In fact, more than 60% of Lm home to the liver as early as 10 min after i.v. infection [2] and protection from listeriosis includes hepatic granuloma formation [47]. Our histopathological demonstration of granulomas in Cyld^−/−^ mice indicates that the increased fibrin production in these animals contributed to the containment of Lm in the hepatic parenchyma. In contrast, WT mice failed to establish granulomas resulting in ubiquitous dissemination of Lm, which caused widespread liver necrosis.

A series of *in vivo* studies identified CYLD as a molecular regulator and link of both innate immune responses including NF-κB-dependent IL-6 production and the coagulation system, i.e. STAT3-dependent fibrin production: (i) neutralization of IL-6 reduced STAT3 activity, fibrin production, and hepatic control of Lm in Cyld^−/−^ mice, (ii) knockdown of STAT3 reduced fibrin production, diminished control of Lm, and reduced serum IL-6 levels in Cyld^−/−^ mice, which is in agreement with Samavati et al. [30], who also observed a STAT3-dependent IL-6 production in LPS-stimulated murine macrophages, and finally (iii) fibrin neutralization by warfarin abolished pathogen control in Cyld^−/−^ mice.

The observation that siRNA-mediated inhibition of CYLD in WT mice increased hepatic p-STAT3 and fibrin levels, diminished hemorrhage and significantly increased survival indicates that inhibition of CYLD might be a therapeutic option in severe listeriosis and potentially other infectious diseases including acute lung injury induced by *S. pneumoniae* [16]. However, treatment with CYLD inhibitors might be limited to acute infections and prolonged CYLD inhibition bears the intrinsic risk of cancer development, since CYLD augments NF-κB-STAT3 crosstalk, which supports development of several neoplasms including hepatocellular carcinoma [48], [49], and Cyld mutations are frequently observed in these tumors [19], [20].

## Materials and Methods

### Animals

Age and sex matched C57BL/6 WT were obtained from Janvier (Le Genest Saint Isle, France) and C57BL/6 Cyld^−/−^ mice were published by us before [23]. All animals were kept under conventional conditions in an isolation facility throughout the experiments.

### Ethics statement

All animal experiments were in compliance with the German animal protection law in a protocol approved by the Landesverwaltungsamt Sachsen-Anhalt (file number: 203.h-42502-2-901, University of Magdeburg).

### Bacterial infection of mice

Lm (EGD strain) was grown in tryptose soy broth and aliquots of log-phase cultures were stored at −80°C. For i.v. infection, fresh log-phase cultures were prepared from frozen stocks and 5×10^5^ Lm diluted in 200 µl sterile pyrogen-free PBS (pH 7.4) were injected. In each experiment, the bacterial dose used for infection was controlled by plating an inoculum on tryptose soy agar and counting colonies after incubation at 37°C for 24 h.

### CFU

To determine CFUs in Lm-infected mice, organs were dissected and homogenised with sterile tissue grinders. Ten-fold serial dilutions of the homogenates were plated on tryptose-soy agar. Bacterial colonies were counted microscopically after incubation at 37°C for 24 h and 48 h.

### Isolation of serum and hepatic leukocytes

Animals were anesthetized with isoflurane and blood was obtained by puncture of the heart with a 25 gauge needle attached to a heparinised 1 ml syringe. After centrifugation, serum was collected and stored at −80°C. Thereafter, mice were intracardially perfused with 0.9% PBS to remove contaminating intravascular leukocytes. Thereafter, liver tissue was minced through a 100 µm cell strainer, and leukocytes were separated by Percoll gradient centrifugation (GE Healthcare, Freiburg, Germany) as described before [14]. Hepatic erythrocytes were lysed with ammonium chloride.

### Histopathology

For immunohistochemistry on frozen sections, mice were perfused intracardially with 0.9% NaCl in methoxyflurane anaesthesia. For histology on paraffin sections, anesthetized mice were perfused with 4% paraformaldehyde in PBS, liver was removed and fixed with 4% paraformaldehyde for 24 h. Paraffin sections (4 µm) were used for periodic acid Schiff (PAS) staining. Immunostaining with antibodies against Lm was performed rabbit α-Lm (BD Biosciences, Heidelberg, Germany) followed by peroxidase-labelled goat α-rabbit immunoglobulin G F(ab′)_2_ fragments (Jackson-Dianova, Hamburg, Germany). Peroxidase reaction products were visualized by 3,3′-diamonobenzidine tetrahydrochloride (Sigma, Deisenhofen, Germany), and H_2_O_2_ was used as the co-substrate. Images were acquired with a Zeiss Axiophot using Zeiss Axioplan objective lenses, a Zeiss Axicam camera and Zeiss Axiovision software (all from Zeiss, Oberkochen, Germany).

### Hepatocyte culture

Mouse liver was perfused with HEPES buffer followed by collagenase solution (Sigma). Isolated hepatocytes were washed with PBS. 1×10^6^ cells were plated on 6 cm dishes in DMEM containing 10% fetal calf serum, 10% non essential amino acids, 1% L-glutamine and a combination of penicillin and streptomycin. The medium was changed after 4 hours. The cells were then cultured in DMEM containing 10% fetal calf serum, 10% non essential amino acids, 1% L-glutamine and a combination of penicillin and streptomycin. The cells were stimulated with 200 ng/ml recombinant IL-6 (PeproTech GmbH, Hamburg, Germany). Cells were harvested at the indicated time points, and proteins were isolated for WB.

### Transfection of hepatocytes

Cultivated hepatocytes (5×10^6^ cells per experimental group) were transiently transfected with HA-tagged ubiquitin in which all lysines were mutated to arginines (HA-ubiquitin-KO, plasmid 17603), ubiquitin with only K63 and mutation of all other lysines to arginines (HA-ubiquitin K63, plasmid 17606) ubiquitin with all lysines present (HA-ubiquitin-WT, plasmid 17608; all from Addgene, Cambridge MA), HA-CYLD WT, HA-CYLD C/S (catalytically inactive CYLD) [18], and MYC-DDK STAT3 (Origene, Rockville, MD) plasmids as indicated using the Lipofectamine 2000 reagent (Invitrogen, San Diego, CA) according to the manufacturer's instructions for 48 hrs. Transfected hepatocytes were stimulated with IL-6 (200 ng/ml, PeproTech) for 1 h.

### Western blot

Cultivated hepatocytes and liver tissue were resuspended in lysis buffer containing 50 mM Tris-HCl (pH 7,4), 5 mM EDTA, 100 mM NaCl, 1% Triton-X-100, 10% glycerol, 10 mM KH_2_PO_4_, 0,5% Na-deoxycholate, 1 mM PSMF, 1 mM NaF, 1 mM Na_4_O_7_P_2_, 1 mM Na_3_VO_4_ and aprotinin, leupeptin, pepstatin (all reagents from Sigma, Taufkirchen, Germany; 1 µg/ml each). Equal amounts of proteins were separated on 10% SDS-polyacrylamide gels, transferred to polyvinylidene fluoride membranes followed by incubation with: α-CYLD (Abcam, Cambridge, UK), α-IκBα, α-phospho-Jak2 (Santa Cruz Biotechnology, Heidelberg, Germany), α-phospho-p65, α-p65, α-phospho-STAT3, α-STAT3, α-phospho-p38 MAPK, α-p38 MAPK, α-PAI-1, α-Jak2, α-ubiquitin K63, α-tubulin, α-HDAC, α-phospho-IκBα, α-GAPDH, α-HA (all from Cell Signaling Technology, Danvers, MA), α-DDK (Origene), and α-β-chain fibrin antibodies (American Diagnostica, Stamford, CT; binds specifically to the β-chain of fibrin but not fibrinogen) [50], [51]. Cytoplasmic and nuclear proteins were isolated with a commercial kit (NE-Per Nuclear and Cytoplasmic Extraction Kit; Thermo Scientific, Waltham, MA). The purity and amount of cytoplasmic and nuclear proteins were controlled by staining for tubulin and HDAC (both antibodies from Cell Signaling), respectively. Blots were developed using an ECL Plus kit (GE Healthcare, Freiburg, Germany). For quantitation of protein intensities by densitometry, WB images were captured using the Intas Chemo Cam Luminescent Image Analysis system (INTAS Science Imaging Instruments, Göttingen, Germany) and analysed with the LabImage 1D software (Kapelan Bio-Imaging Solutions, Leipzig, Germany).

### Immunoprecipitation

Unstimulated and IL-6-stimulated (200 ng/ml) mouse hepatocytes were lysed on ice as described before. In a pre-clearing phase, Sepharose G beads (GE Healthcare Europe GmbH, Munich, Germany) were incubated for 30 min with cell lysates under continuous shaking at 4°C. The beads were removed by centrifugation and equal amounts of lysates were incubated with α-STAT3, α-DDK, and α-HA antibodies, respectively, at 4°C overnight. The immune complex was captured by incubating with Sepharose G beads overnight at 4°C. The beads were then washed 3 times with PBS by centrifugation. The pellet containing Sepharose G immune complexes was suspended in buffer 2 (SDS, 1 M pH 6,8 Tris, glycerol; 2-mercaptoethanol) and boiled at 100°C for 5 min. After centrifugation, the supernatant was used for WB. Controls included beads incubated with cell lysate without capturing antibody (B+L) and beads plus antibody without lysates (B+A).

### Reverse transcription-PCR (RT-PCR)

Isolation of mRNA from the livers and spleens of uninfected and Lm-infected mice was performed with an RNAeasy kit (Qiagen, Hilden, Germany). The SuperScript reverse transcriptase kit with oligo(dT) primers (Invitrogen) was used to transcribe mRNA into cDNA. Quantitative RT-PCR for Cyld, IL-6, IFN-γ, NOX2, and hypoxanthine phosphoribosyltransferase (HPRT) was performed with cDNA from C57BL/6 WT and C57BL/6 Cyld^−/−^ mice and the respective Taqman gene expression assay (Applied Biosystems, Darmstadt, Germany). Amplification was performed with a GeneAmp 5700 sequence detection system (Applied Biosystems). Quantitation was performed with the sequence detector software SDS (version 2.1; Applied Biosystems), according to the ΔΔ*C_T_* threshold cycle (*C_T_*) method with HPRT as the housekeeping gene [52]. Data are expressed as the increase in the level of mRNA expression in infected mice over that in uninfected controls of the respective mouse strain. All primers and probes were obtained from Applied Biosystems.

### Determination of AST and ALT

Liver enzymes (ALT, AST) were measured according to recommendation of the International Federation of Clinical Chemistry using commercial tests (Roche Diagnostics, Mannheim, Germany; on the Modular platform) with pyridoxal phosphate activation at 37°C and measurement on the Cobas Modular platform (Roche, Mannheim, Germany).

### Bone marrow-derived macrophages (BMDM)

Femur and tibia were aseptically removed from mice, the bone ends were cut, and the bone marrow cavity was flushed with HBSS. The resulting cell suspension was washed twice and cultured in petri dishes with DMEM supplemented with 10% FCS, 50 U/ml penicillin/streptomycin, 1% non-essential amino acids, 1% glutamine, 20 ng/ml M-CSF, and 50 µM 2-mercaptoethanol for 3 days. Medium was changed every 3 days and non-adherent cells were removed by washing the dishes. After 6 days, adherent BMDMs were harvested and used for experiments.

### *In vitro* infection of BMDMs with Lm

BMDMs were stimulated with IFN-γ (100 U/ml, PeproTech) overnight, before infection with Lm at an MOI of 5∶1 in DMEM supplemented with 10% FCS, 50 U/ml penicillin/streptomycin, 1% non-essential amino acids, 1% glutamine and 50 µM 2-mercaptoethanol. After 1 h of infection, 30 µg/ml gentamicin (Sigma) was added to kill extracellular bacteria for additional 3 hours. Thereafter, infected BMDMs were washed in PBS, resuspended in DMEM supplemented with 15 µg/ml gentamicin and cultured for the indicated time points. For inhibition of NF-κB, BMDMs were treated with IKK inhibitor VII (10 µM for 4 h followed by 1 µM for 20 h; Calbiochem, Darmstadt, Germany). Proteins of BMDMs were isolated as described for hepatocytes at the indicated time points of infection and used for WB. The supernatant of infected BMDMs was harvested 24 h p.i. and analysed for NO and IL-6.

### ROS assay

ROS was determined in cultivated Lm-infected macrophages using a total ROS detection kit for flow cytometry according to the manufacturer's protocol (ENZO Life Sciences, Farmingdale, U.S.A).

### IL-6 neutralization

In IL-6 neutralization experiments, mice were treated with 0.5 mg neutralizing α-IL-6 antibody i.p. (clone MP5-20F3, rat IgG1; ATCC) or isotype mAb (α-rat IgG1; Sigma), respectively. Antibodies were applied 24 h prior to Lm infection.

### Warfarin treatment

Warfarin (3-(α-acetonylbenzyl)-4-hydroxycoumarin; 2 mg/l, Sigma) was added to drinking water of mice beginning 3 days prior to infection. Warfarin containing drinking was changed every 48 h and treatment was continued until day 10 after infection.

### Flow cytometry and cytometric bead assay

Leukocytes isolated from the liver were analysed by flow cytometry on a FACS Canto II with Cell Quest software (both from BD Biosciences). Cells were stained with α-CD45 in combination with α-CD4 and CD3 for CD4 T cells, α-CD8 and CD3 for CD8 T cells, α-NK1.1 and CD3 for NK cells, F4/80 and CD11b for macrophages, CD11c and CD11b for dendritic cells, Ly6G and Ly6C for inflammatory monocytes, and Ly6G and CD11b for granulocytes. Control staining was performed with isotype-matched control antibodies. All antibodies were obtained from BD Biosciences. Cytokine levels in serum were analysed by flow cytometry using the Cytometric Bead Assay (CBA) from BD Biosciences (San Jose, CA) using the manufacturer's protocol and FCAP Array (version 3, BD Biosciences) software.

### *In vivo* siRNA treatment

CYLD and STAT3 siRNA were obtained from Ambion (CA, USA). A 1.5 mg/ml siRNA solution was prepared by mixing 250 µl of CYLD and STAT3 siRNA stock solution (3 mg/ml), respectively, with 250 µl of complexation buffer. Invivofectamine 2.0 reagent (Invitrogen) was warmed to room temperature and 500 µl Invivofectamine was added to the siRNA solutions. The Invivofectamine-siRNA duplex mixture was incubated at 50°C for 30 min. The mixture was dialysed with PBS using Float-A-Lyzer (Company) for 1 h. 200 µl of siRNA with a final concentration of 7 mg siRNA/kg mouse siRNA was injected into the tail vein 24 to 48 h before i.v. infection with Lm.

### Statistics

Statistical significance was determined using the two-tailed Student *t* test or nonparametric Mann-Whitney rank sum test using Statistica 5 software (StatSoft, OK, USA). All experiments were performed at least twice. *P* values of <0.05 were considered significant.

## Supporting Information

Figure S1 **Flow cytometric analysis of hepatic leukocytes.** Hepatic leukocytes were isolated from Lm-infected mice at day 5 p.i. and analysed by flow cytometry. Leukocytes were first gated using FSC-A and SSC-A followed by gating on CD45^+^ cells in combination with SSC-A. CD45^+^ gated cells were further analysed and dot plots for CD8^+^ CD3^+^ T cells, CD11b^+^ Ly6G^high^ granulocytes, and F4/80^+^ CD11b^+^ macrophages, respectively, are shown. The percentage of positive cells is shown in the upper right quadrant of the dot plots. Representative dot plots from 1 out of 5 mice per experimental group are shown.
(TIF)
