# Supplementary figures and images for: CYLD Enhances Severe Listeriosis by Impairing IL-6/STAT3-Dependent Fibrin Production
Source: PLoS Pathog. 2013 Jun 27;9(6):e1003455. doi: 10.1371/journal.ppat.1003455 (PMC3695090; doi:10.1371/journal.ppat.1003455)

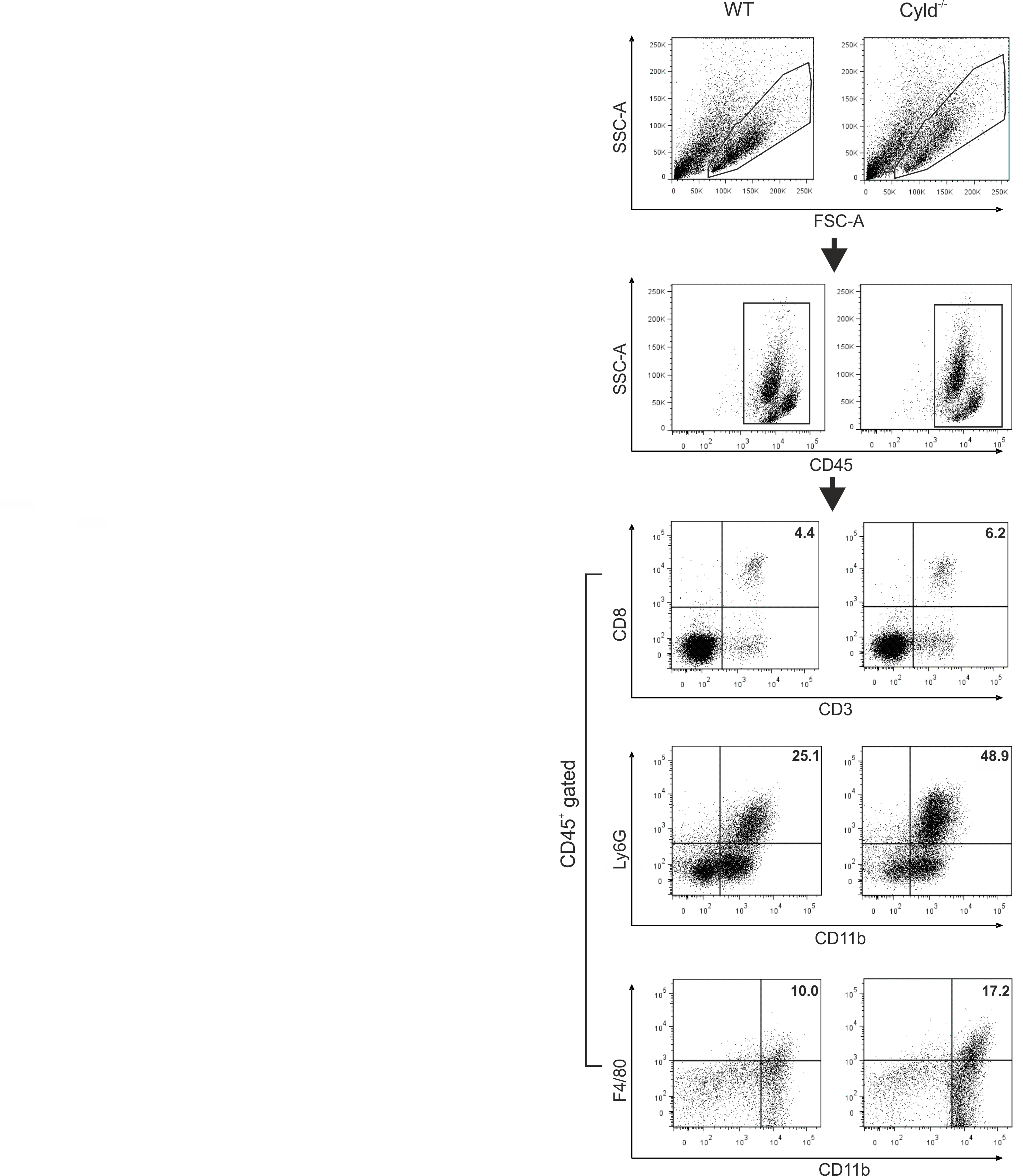

Supplement: Figure S1 — Flow cytometric analysis of hepatic leukocytes. Hepatic leukocytes were isolated from Lm-infected mice at day 5 p.i. and analysed by flow cytometry. Leukocytes were first gated using FSC-A and SSC-A followed by gating on CD45+ cells in combination with SSC-A. CD45+ gated cells were further analysed and dot plots for CD8+ CD3+ T cells, CD11b+ Ly6Ghigh granulocytes, and F4/80+ CD11b+ macrophages, respectively, are shown. The percentage of positive cells is shown in the upper right quadrant of the dot plots. Representative dot plots from 1 out of 5 mice per experimental group are shown. (TIF) [file ppat.1003455.s001.tif]
